# Genome-wide markers reveal a complex evolutionary history involving divergence and introgression in the Abert’s squirrel (*Sciurus aberti*) species group

**DOI:** 10.1186/s12862-018-1248-4

**Published:** 2018-09-12

**Authors:** Jeremy M. Bono, Helen K. Pigage, Peter J. Wettstein, Stephanie A. Prosser, Jon C. Pigage

**Affiliations:** 1 Department of Biology, University of Colorado Colorado Springs, Colorado Springs, CO 80918 USA; 2 Department of Surgery, Mayo Clinic, 200 First St. SW, Rochester, MN 55905 USA

**Keywords:** Admixture, Divergence with gene flow, Mito-nuclear discordance, Mitochondrial capture, Hybridization, Ponderosa pine

## Abstract

**Background:**

Genetic introgression between divergent lineages is now considered more common than previously appreciated, with potentially important consequences for adaptation and speciation. Introgression is often asymmetric between populations and patterns can vary for different types of loci (nuclear vs. organellar), complicating phylogeographic reconstruction. The taxonomy of the ecologically specialized Abert’s squirrel species group has been controversial, and previous studies based on mitochondrial data have not fully resolved the evolutionary relationships among populations. Moreover, while these studies identified potential areas of secondary contact between divergent lineages, the possibility for introgression has not been tested.

**Results:**

We used RAD-seq to unravel the complex evolutionary history of the Abert’s squirrel species group. Although some of our findings reinforce inferences based on mitochondrial data, we also find significant areas of discordance. Discordant signals generally arise from previously undetected introgression between divergent populations that differentially affected variation at mitochondrial and nuclear loci. Most notably, our results support earlier claims (disputed by mitochondrial data) that *S. aberti kaibabensis*, found only on the north rim of the Grand Canyon, is highly divergent from other populations. However, we also detected introgression of *S. aberti kaibabensis* DNA into other *S. aberti* populations, which likely accounts for the previously inferred close genetic relationship between this population and those south of the Grand Canyon.

**Conclusions:**

Overall, the evolutionary history of Abert’s squirrels appears to be shaped largely by divergence during periods of habitat isolation. However, we also found evidence for interbreeding during periods of secondary contact resulting in introgression, with variable effects on mitochondrial and nuclear markers. Our results support the emerging view that populations often diversify under scenarios involving both divergence in isolation and gene flow during secondary contact, and highlight the value of genome-wide datasets for resolving such complex evolutionary histories.

**Electronic supplementary material:**

The online version of this article (10.1186/s12862-018-1248-4) contains supplementary material, which is available to authorized users.

## Background

The evolutionary histories of many species are characterized by repeated range contractions and expansions, leading to periods of isolation and subsequent secondary contact between divergent lineages [1, 2]. Interbreeding during secondary contact can result in genetic introgression, which appears to be more common than previously assumed and has significant consequences for the diversification process [3–6]. Patterns of introgression are often highly variable, involving asymmetry in the direction of gene flow among populations, and also differences in the amount of introgression across distinct genomes (i.e. organellar or nuclear) or genomic regions [3].

In the past, phylogeographic reconstruction has relied extensively on the use of mitochondrial DNA. More recently, the inclusion of nuclear loci has become more common, particularly with the advent of next-generation sequencing technologies that enable genome-wide sampling [7–9]. As more such datasets are generated, discordance between mitochondrial and nuclear genomes has been detected with increasing regularity [3]. In many cases, this discordance is explained by differential patterns of introgression for mitochondrial and nuclear loci, with introgression of mitochondrial haplotypes seemingly occurring more easily than nuclear loci [3, 10, 11]. In extreme cases, the mitochondrial genome of one lineage has completely replaced that of another, as, for example, observed in chipmunks from the genus *Tamias* [12]. Complete mitochondrial capture could be explained by positive selection on favorable mitochondrial haplotypes or genetic drift coupled with specific demographic scenarios such as range expansion or sex-related asymmetries in gene flow [3, 11, 13, 14].

Abert’s squirrels (*Sciurus aberti*) are endemic to the Rocky Mountain region of Colorado, Arizona, and New Mexico in the United States, and the Sierra Madre Occidental, in northern Mexico. In the Rocky Mountain region, they are considered habitat specialists, relying on ponderosa pine (*Pinus ponderosa var. scopulorum*) for nesting sites and feeding almost exclusively on hypogeous fungi associated with tree root systems, seeds, and inner bark of terminal twigs [15–17]. This ecological dependence has resulted in a distribution that is almost entirely coincident with ponderosa pine in the Rocky Mountains, although squirrels are absent from many areas occupied by ponderosa pine and have persisted in other areas without extensive stands of ponderosa pine following human introductions [18, 19]. Less is known about associations in the Sierra Madre Occidental of Mexico, but squirrels are found in forests occupied by pine species that were once considered varieties of ponderosa pine (e.g. *P. arizonica*). Within the range of Abert’s squirrels, pine forests are mainly restricted to montane environments (between ≈1800–2600 m), forming a patchwork of “islands” isolated by broad barriers of unsuitable habitat. Current habitat isolation, coupled with past range contractions and expansions associated with glaciation cycles, has resulted in considerable genetic and morphological divergence among populations [20–22].

The Abert’s squirrel complex has been the subject of numerous taxonomic debates resulting in several revisions over the years. For example, the population on the Kaibab Plateau of Arizona is separated from the closest neighboring populations by the Grand Canyon, and was once considered a textbook example of allopatric speciation due to vicariance [23–25]. *Sciurus kaibabensis* was later relegated to subspecies status due to a lack of significant morphological differentiation from populations south of the Grand Canyon outside of conspicuous differences in pelage color [20]. While previous taxonomic schemes have included as many as nine subspecies of *S. aberti*, the most recent formal taxonomic revision of the group includes a total of six [20]. Subsequent genetic studies based on mitochondrial DNA (*cytochrome b* and data from restriction profiles across the entire mitochondrial genome), supported some, but not all, of these designations [21, 22]. Notably, these studies supported the close relationship between *S. aberti kaibabensis* and populations of *S. aberti aberti* south of the Grand Canyon, with minimal genetic divergence between these populations evident at mitochondrial loci. Based on these data, it was reasonably concluded that *S. aberti kaibabensis* likely colonized the north rim of the Grand Canyon only recently from source populations on the south side of the canyon [21, 22]. These studies further revealed a deep split between these western populations (*S. aberti kaibabensis* and *S aberti aberti* from Arizona) and populations further east in New Mexico and Colorado. Interestingly, *S. aberti chuscensis*, found only in the Chuska mountain range on the border of New Mexico and Arizona, included individuals carrying mitochondrial haplotypes from both western and eastern clades [21, 22]. The presence of these divergent haplotypes, coupled with the location of this population near the center of an area of unsuitable habitat separating the western and eastern clades, suggested a scenario of secondary contact between divergent eastern and western lineages in this region. Since these data were derived from haploid non-recombining mitochondrial DNA samples, it was not possible to infer whether genetic admixture between these lineages has subsequently occurred in this location. These previous studies also failed to fully resolve the relationship between the most southern populations in Mexico (*S. aberti barberi* and *S. aberti durangi*) and the populations in the United States. The study based on *cytb* sequences suggested these populations were the first to diverge from the others [21], as might be expected if *S. aberti* initially expanded from southern glacial refugia. However, the study based on mitochondrial restriction profiles suggested that these populations were more closely-related to members of the eastern clade than to those from the western clade [22].

Abert’s squirrels represent an exciting system in which to investigate how patterns of divergence and admixture resulting from historical range shifts impact genetic diversity and differentiation. In this study, we use genome-wide single nucleotide polymorphisms (SNPs) generated from Restriction Associated DNA sequencing (RAD-seq data) to further resolve the evolutionary history of the complex of *S. aberti* species.

## Results

### Population structure and genetic diversity

**Fig. 1:**
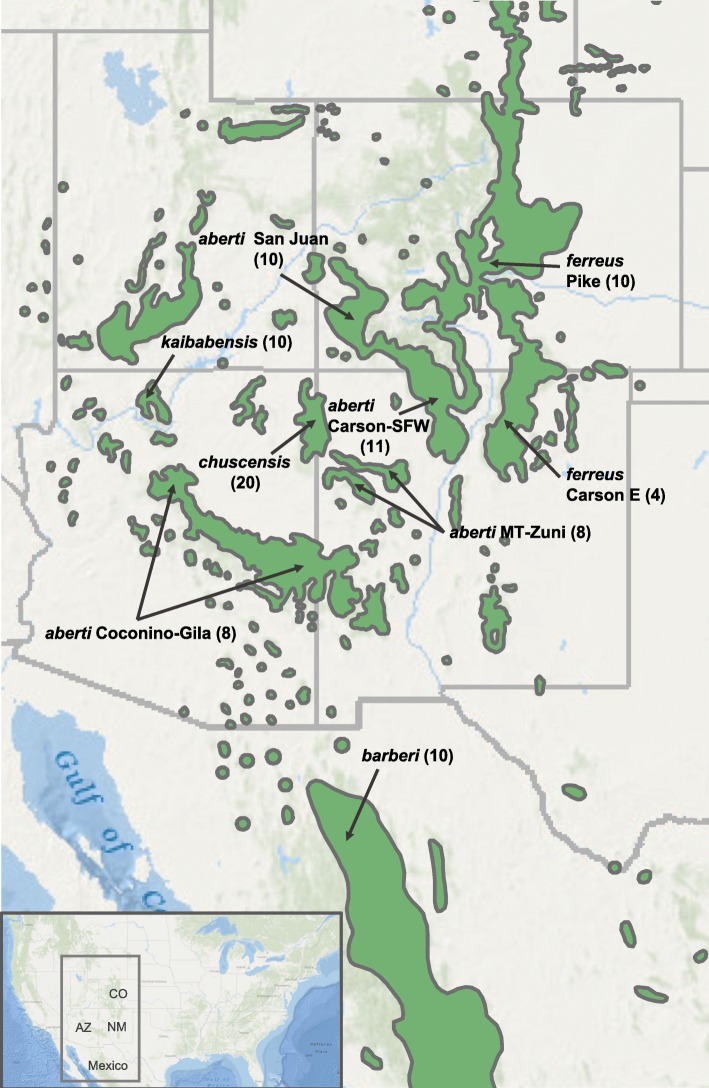
*S. aberti* approximate collecting locations with samples sizes given in parentheses. The distribution of ponderosa pine (or closely related species in Mexico) is shaded in green. MT = Mount Taylor; SFW = Santa Fe West; E = East; AZ = Arizona; NM = New Mexico; CO = Colorado

**Fig. 2:**
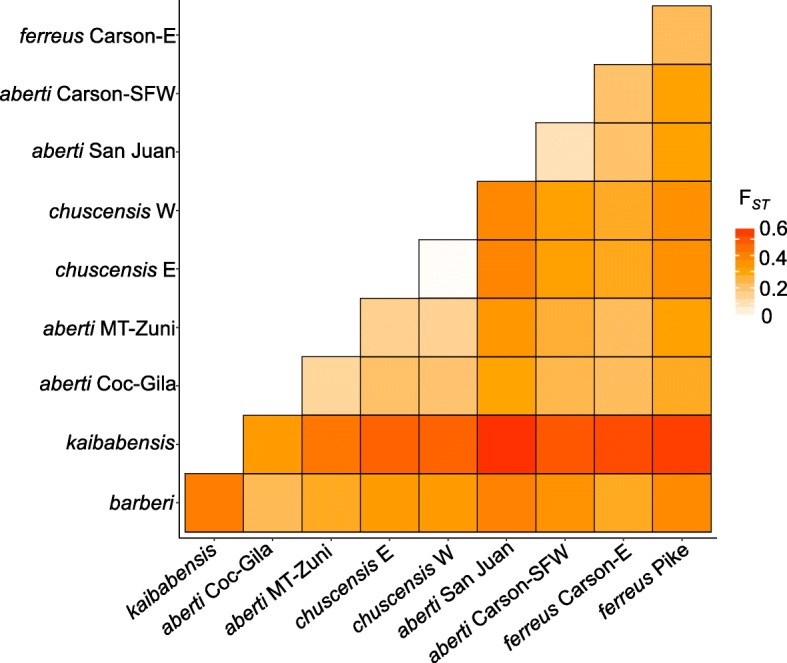
Heatmap showing Weir and Cockerham’s pairwise *F*_ST_ values calculated using genome-wide SNPs

We used RAD-seq to identify SNPs for genetic analyses of samples collected from across the range of *S. aberti* (Fig. 1) and one outgroup sample (*S. griseus*). Population structure analysis using Weir and Cockerham’s F_*ST*_ revealed substantial genetic structure among *S. aberti* populations collected from different localities with the F_*ST*_ overall being 0.339 (CI: 0.336–0.342). Pairwise F_*ST*_ values were generally quite high with the exception the comparison of *S. aberti chuscensis* samples carrying the different mitochondrial haplotypes (Fig. 2; Additional file 1). The largest values were between *S. aberti kaibabensis* and all other populations (range: 0.332–0.568), and *S. aberti barberi* and all other populations (range: 0.228–0.413). Among other comparisons, the lowest value was between *S. aberti ferreus* from San Juan and Carson-SFW (0.098) and the highest values were between the *S. aberti chuscensis* groups and *S. aberti aberti* from San Juan (0.431 and 0.383).

**Fig. 3:**
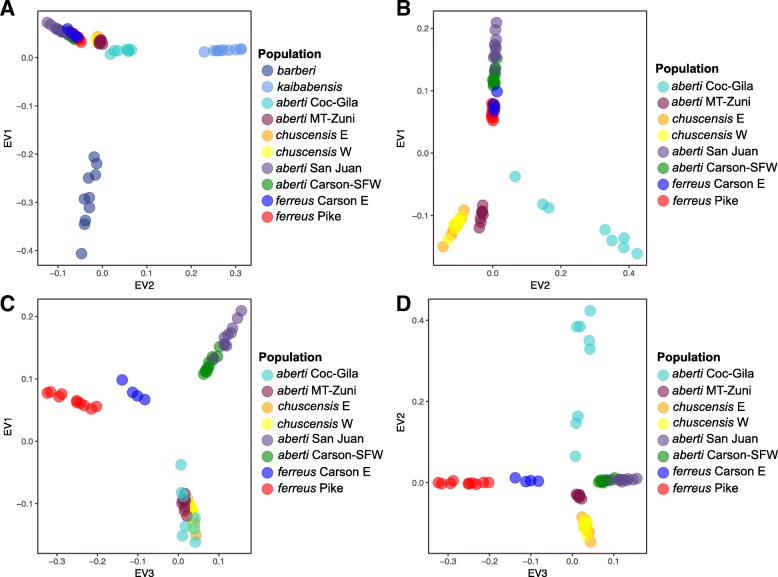
Principal Components Analysis plots based on genome-wide SNPs. (**a**) Plot of the first two eigenvectors with all populations included, (**b**) plot of the first two eigenvectors with *S. aberti barberi* and *S. aberti kaibabensis* removed from the analysis, (**c**) plot of the first and third eigenvectors with *S. aberti barberi* and *S. aberti kaibabensis* removed from the analysis, (**d**) plot of the second and third eigenvectors with *S. aberti barberi* and *S. aberti kaibabensis* removed from the analysis

The first three eigenvectors from the Principal components analysis (PCA) explained 7.7, 4.1, and 3.3% of variation, respectively, and largely separated all the groups except for the samples carrying divergent mitochondrial haplotypes in *S. chuscensis* (Fig. 3a). In line with *F*-statistics, *S. aberti barberi* and *S. aberti kaibabensis* were the most divergent populations, being clearly separated from all other samples by the first two eigenvectors. Because the magnitude of this divergence made it difficult to visualize separation of the other populations, we removed *S. aberti barberi* and *S. aberti kaibabensis* and ran the analysis again (Figs. 3b, c, and d). Eigenvectors one and two clearly separated western (*S. aberti aberti* Coconino-Gila/MT-Zuni and *S. chuscensis*) and eastern (*S. aberti aberti* Carson-SFW/San Juan, and *S. aberti ferreus*) samples, and distinguished groups within the western samples. The third eigenvector separated the eastern samples, although there was some overlap among samples from San Juan and Carson-SFW. Samples from Carson E were approximately intermediate between San Juan/Carson-SFW and Pike.

**Fig. 4:**
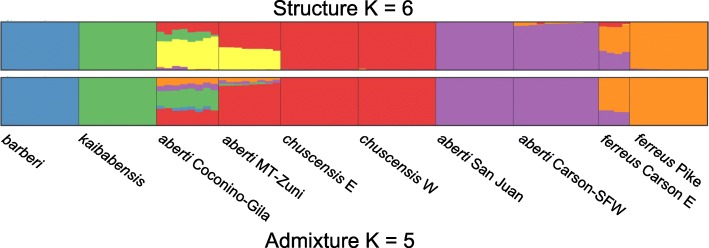
Structure (top) and Admixture (bottom) bar plots showing the portion of ancestry drawn from each genetic cluster for optimal values of *K*

Genetic clustering by ADMIXTURE and STRUCTURE also indicated significant population structure, with *K* = 5 being chosen as optimal by the CV procedure of ADMIXTURE and *K* = 6 identified as optimal for the STRUCTURE analysis based on Evanno’s method (Fig. 4). Although the number of optimal genetic clusters was different for the two methods, overall there was broad agreement between them, with members of most populations drawing ancestry mainly from a single genetic cluster. Moreover, both methods suggested that the *S. aberti aberti* Coconino-Gila population, the *S. aberti aberti* MT-Zuni population, and the *S. aberti ferreus* Carson E population had a mixed pattern of ancestry. The exact pattern of admixture for these populations differed, however. For example, although both methods indicated that *S. aberti aberti* Coconino-Gila population and the *S. aberti aberti* MT-Zuni population were admixed, the STRUCTURE analysis identified a separate genetic cluster unique to these two populations and little ancestry from clusters mainly associated with eastern populations. In contrast, the ADMIXTURE analysis indicated that the *S. aberti aberti* Coconino-Gila population had ancestry from all genetic clusters, while *S. aberti aberti* MT-Zuni lacked any ancestry from the genetic cluster mainly associated with *S. aberti barberi*. Notably, both methods showed that individuals from *S. aberti aberti* Coconino-Gila had substantial ancestry from the genetic cluster associated with *S. aberti kaibabensis*, and the ADMIXTURE results indicated some ancestry from this cluster in *S. aberti aberti* MT-Zuni as well. Both methods indicated mixed ancestry for individuals from the *S. aberti ferreus* Carson E population, with evidence for ancestry from genetic clusters associated with *S. aberti ferreus* Pike and *S. aberti aberti* San Juan/Carson-SFW.

**Fig. 5:**
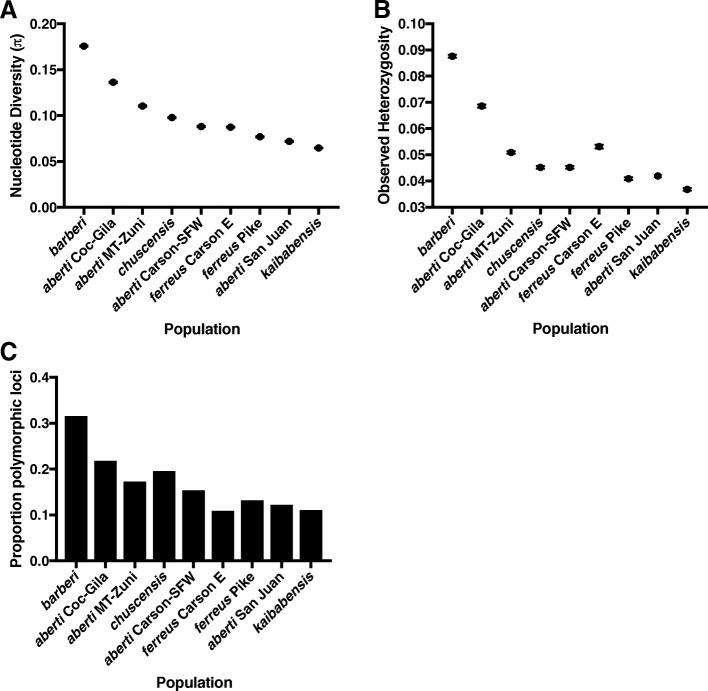
Genetic diversity plots. (**a**) Nucleotide diversity (π), (**b**) observed heterozygosity, and (**c**) proportion of polymorphic loci

All measures of genetic diversity were highest in the *S. aberti barberi* population from Mexico, with *S. aberti aberti* Coconino-Gila exhibiting the next highest levels (Fig. 5). Conversely, *S. aberti kaibabensis* generally exhibited the lowest genetic diversity. In general, genetic diversity tended to be lower in eastern populations than in western populations*.* Although we acknowledge that RAD-seq can lead to underestimates of genetic diversity due to the loss of restriction sites [7, 26, 27], our main interest was to compare relative levels of diversity among populations. These relative estimates do not appear to be greatly affected by allelic dropout, as loss of restriction sites should be most common in the most divergent populations, leading to underestimates of diversity in these samples. In contrast to this expectation, *S. aberti barberi*, which was highly divergent from all other populations, also had the highest relative diversity.

### Evolutionary relationships and introgression among populations

**Fig. 6:**
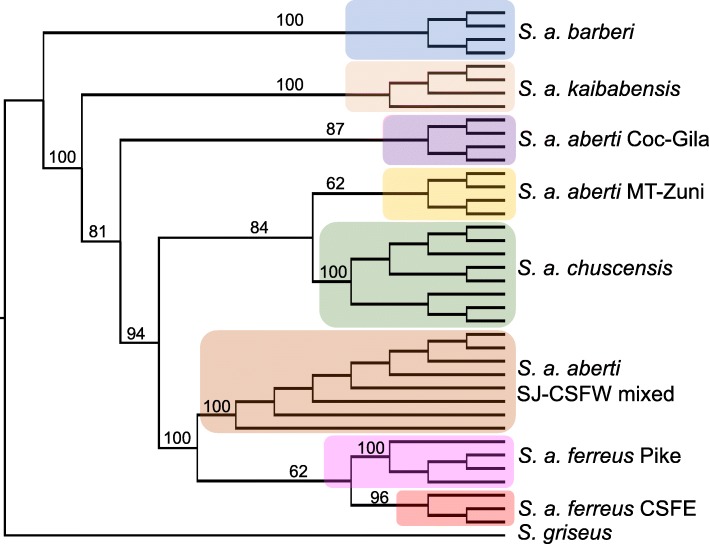
Maximum-likelihood tree with bootstrap support values for major clades produced by RAXML-NG (GTR model with ascertainment bias correction)

**Fig. 7:**
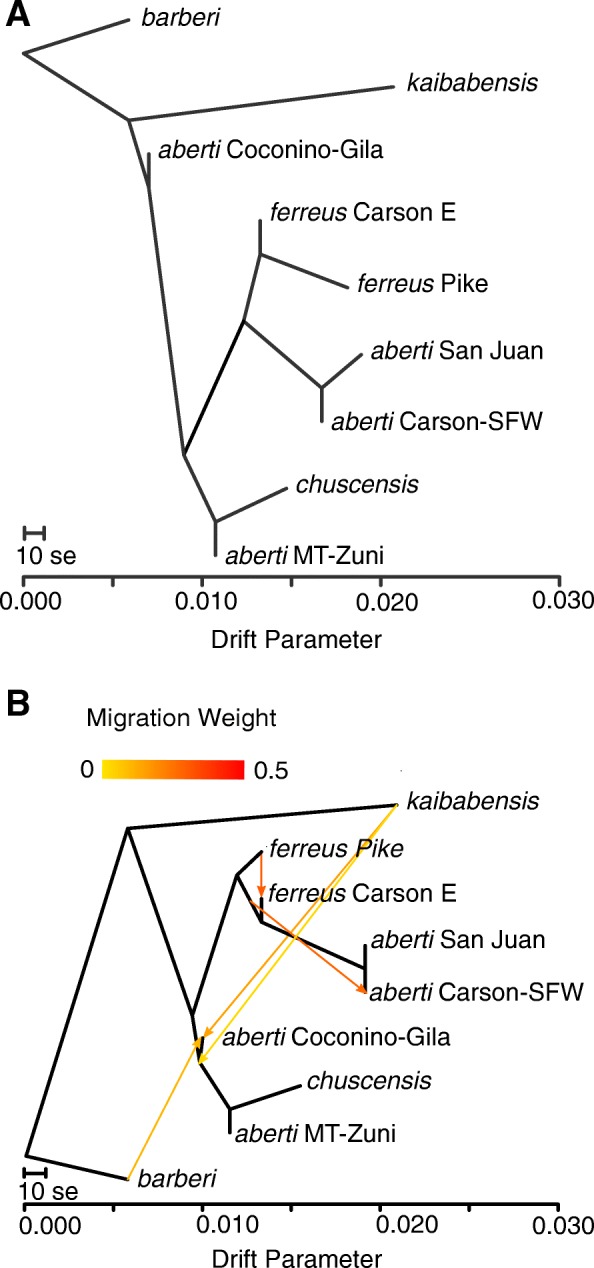
(**a**) Maximum-likelihood tree produced by TREEMIX with no migration, (**b**) maximum likelihood tree produced by TREEMIX with five migration edges

The best maximum-likelihood trees produced by RAXML-NG using either GTR or TVM nucleotide substitution models exhibited the same topology with respect to the sampled populations (results from the GTR model are shown in Fig. 6). The tree gives strong support for monophyly of all groups except for *S. aberti aberti* Carson-SFW/*S. aberti aberti* San Juan, which were intermingled, and *S. aberti chuscensis* samples carrying the two haplotype lineages, which were also intermingled (Fig. 6). This analysis also identified *S. aberti barberi* as the earliest branching lineage in the group. We do not present information on branch lengths since previous studies have shown that they can be unreliable for concatenated SNP datasets [28]. Based on the RAXML-NG tree, we designated *S. aberti barberi* as the outgroup in the TREEMIX analysis. The maximum-likelihood tree with no migration produced by TREEMIX showed the same relationships among *S. aberti* populations as the RAXML-NG tree (Fig. 7a). We added migration events to the tree in a step-wise manner until additional edges were no longer statistically significant, which resulted in a total of five migration edges (Table 1). The topology of the final tree with five migration edges differed from the original tree with no migration, as *S. aberti aberti* Coconino-Gila moved into a clade with *S. aberti aberti* MT-Zuni and *S. aberti chuscensis*, and the position of *S. aberti ferreus* Carson E shifted to be more closely related to *S. aberti aberti* San Juan/Carson-SFW than *S. aberti ferreus* Pike (Fig. 7b). We treat the placement of these populations somewhat cautiously since TREEMIX does not assign confidence to the tree topology. However, we also note that these relationships are generally consistent with other analyses (e.g. PCA and genetic clustering). In general, the migration edges inferred by TREEMIX were supported by patterns of admixture suggested by at least one of genetic clustering analyses. To more robustly examine the significance of each migration edge we conducted a series of four-population tests (Table 1). We did not test the third edge (population equally related to *ferreus* Carson E, *ferreus* Carson-SFW, *ferreus* San Juan - > *ferreus* Carson-SFW) since the source population was not sampled. These tests generally supported the validity of the migration edges inferred by TREEMIX. The one exception involved the introgression from *S. aberti kaibabensis* into the common ancestor of *S. aberti aberti* MT-Zuni and *S. aberti chuscensis*, which was only partially supported. While the four-population test for introgression of *S. aberti kaibabensis* into *S. aberti aberti* MT-Zuni was supported, the test for introgression into *S. aberti chuscensis* was marginally non-significant (Table 1).

**Table 1:** Migration edges inferred by TREEMIX and results of four population tests

Migration edge added	Migration weight in final model	Variance explained	*F*_*4*_ Test Tree	Observed *f*_*4*_	*P*-value
No migration	–	97.8%	–	–	–
*kaibabensis - > aberti* Coc-Gila	0.146	98.6%	(*ferreus Pike*, *kaibabensis; chuscensis*, *aberti* Coc-Gila)	0.01514	< 0.00001
*ferreus* Pike - > *ferreus* Carson E	0.303	99.3%	(*ferreus* Pike, *chuscensis*; *ferreus* Carson E, *aberti* San Juan)	0.01082	< 0.00001
equally related to *ferreus* Carson E, *aberti* Carson-SFW, *aberti* San Juan - > *aberti* Carson-SFW	0.279	99.6%	–		
*barberi* - > *aberti* Coc-Gila	0.101	99.8%	(*aberti* Coc-Gila, *chuscensis*; *barberi*, *ferreus* Pike)	0.00706	< 0.00001
*kaibabensis* - > common ancestor of *chuscensis*, *aberti* MT-Zuni, and *aberti* Coc-Gila	0.030	99.9%	(*barberi*, *kaibabensis*; *ferreus* Pike, *aberti* MT-Zuni)	0.00156	0.0160
			(*barberi*, *kaibabensis*; *ferreus* Pike, *chuscensis*)	0.00116	0.0720

### Mitochondrial introgression: Construction of *cytb* haplotype network and haplotyping

**Fig. 8:**
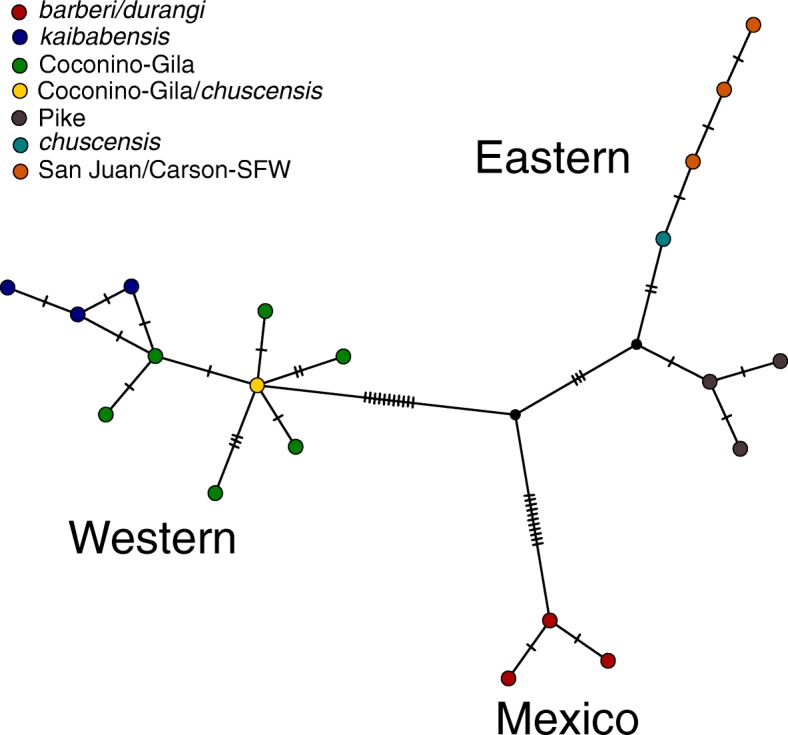
TCS haplotype network generated from *cytb* sequences

As expected based on the previous study that generated the *cytb* sequence data, the TCS haplotype network showed clear divisions among haplotypes from Mexico (*S. aberti barberbi* and *S. aberti durangi*), and what were previously considered western and eastern haplotype lineages (Fig. 8). The Mexican samples were separated from the eastern haplogroup by a minimum of 14 mutational steps and the western network by a minimum of 22 mutational steps. Western and eastern haplotypes were also highly divergent with a minimum of 14 mutational steps separating them. To provide an informal metric to compare relative levels of divergence between populations using mitochondrial DNA and genome-wide SNPs, we used the *populations* module in STACKS to generate an alignment of loci present at least 50% of individuals in all populations that exhibited fixed differences between populations (145 loci). In this alignment, *S. aberti barberi* and *S. aberti kaibabensis* were about equally divergent from other populations (e.g. 53.1 and 51.7% identity between these respective populations and the *S. aberti aberti* Coconino-Gila population). In contrast, pairwise identity between *S. aberti aberti* Coconino-Gila and all other populations was uniformly high (e.g. 98.6% between this population and *S. aberti ferreus* Pike). Thus, while the mitochondrial data indicate that representatives from eastern and western haplogroups (e.g. *S. aberti ferreus* Pike and *S. aberti aberti* Coconino-Gila) are as divergent from one another as they are from Mexican samples, the genome-wide data reveal much less divergence between eastern and western samples than exists between either of these groups and Mexican samples.

*Cytb* haplotyping of additional samples suggested that haplotypes from the western lineage are fixed in the *S. aberti aberti* Coconino-Gila (*N* = 44) and Cibola (*N* = 11) populations and in *S. aberti kaibabensis* (*N* = 47), as all additional tested samples had western haplotypes (Additional file 2). In contrast, *S. aberti chuscensis* and MT-Zuni samples had individuals carrying both haplotypes (26 western and 27 eastern for *S. chuscensis*; one western and six eastern for MT-Zuni; Additional file 2).

## Discussion

### Mito-nuclear discordance resulting from asymmetric patterns of introgression

Comparison between the results of our genome-wide analysis and previous analyses of mitochondrial datasets reveals extensive mito-nuclear discordance that complicates interpretation of the evolutionary history of the *S. aberti* complex. Most notably, previous genetic studies had suggested a close relationship between *S. aberti kaibenesis* and *S. aberti aberti* from Arizona, which were highly divergent from eastern populations in Colorado and New Mexico. Moreover, these studies suggested a point of secondary contact between divergent eastern and western lineages within the range of *S. aberti chuscensis* [21, 22]. Our larger genome-wide analysis does not support these scenarios, instead indicating extensive mitochondrial introgression between divergent populations misled previous attempts to piece together the complex evolutionary history of *S. aberti*.

While *S. aberti kaibabensis* has been considered everything from a separate species to a recent offshoot of *S. aberti* populations south of the Grand Canyon [20, 22, 23], multiple types of analyses presented here show that this subspecies is highly diverged from all other *S. aberti* subspecies. However, the data also suggest that *S. aberti kaibabensis* DNA has introgressed into populations of *S. aberti aberti* and *S. aberti chuscensis* in the region of Arizona and western New Mexico. Two migration events were identified, with introgression of *S. aberti kaiabensis* DNA directly into the *S. aberti aberti* Coconino-Gila population, and into the ancestors of *S. aberti aberti* Coconino-Gila, *S. aberti aberti* MT-Zuni, and *S. aberti chuscensis*. Altogether, the results imply that there have likely been multiple periods of secondary contact and admixture between *S. aberti kaibabensis* and other populations in the western part of *S. aberti’s* range. Interestingly, all the evidence indicates that introgression was unidirectional, as no traces of ancestry from other populations are detectable in *S. aberti kaibabensis*. Although the explanation for this is unclear, such a pattern can arise when interbreeding occurs between a resident population and an expanding immigrant population, as introgressed alleles quickly rise in frequency during population expansion [14, 29]. This seems plausible here given that periods of secondary contact likely arose as populations expanded from more restricted ranges during interglacial cycles.

Putting together these results with those from earlier studies, we propose that the previously inferred close relationship between *S. aberti kaibabensis* and *S. aberti aberti* from Arizona [21, 22] likely reflects introgression of mitochondrial DNA with complete fixation of *S. aberti kaibabensis* mitochondrial haplotypes in *S. aberti aberti* from Arizona. While incomplete lineage sorting of ancestral mitochondrial polymorphisms could be an alternative explanation to introgression, we conclude that introgression is the most parsimonious explanation in light of strong evidence for interbreeding between the populations from genome-wide data. Although introgression in either direction would theoretically lead to inference of a close relationship between these populations, several lines of evidence suggest that *S. aberti aberti* captured mitochondrial DNA from *S. aberti kaibabensis* rather than the other way around. First, this scenario is most consistent with the pattern of introgression evident in genome-wide data, with unidirectional movement of *S. aberti kaibabensis* alleles into other western *S. aberti* populations. In addition, the relatively high level of divergence between western mitochondrial haplotypes and those from eastern populations and Mexico suggests that the western haplotype group most likely traces its origins to *S. aberti kaibabensis*. For example, the genome-wide dataset demonstrates that *S. aberti barberi* and *S. aberti kaibabensis* are highly divergent from all other populations, and the magnitude of this divergence is similar. In contrast, divergence among other populations is much lower, even for those representing the most extreme eastern and western regions occupied by *S. aberti* (e.g. *S. aberti aberti* Coconino-Gila and *S. aberti ferreus*). The fact that mitochondrial haplotypes from the western haplogroup are as divergent from eastern haplotypes as they are from the *S. aberti barberi* haplogroup is therefore most easily explained if ancestral haplotypes originated in *S. aberti kaibabensis*. Under the alternative scenario, the high level of divergence between eastern and western haplotypes, on par with divergence from *S. aberti barberi*, would be difficult to explain. Finally, introgression of *S. aberti kaibabensis* haplotypes into other *S. aberti* populations would provide an explanation for the presence of divergent mitochondrial haplotypes in *S. aberti chuscensis* and *S. aberti aberti* MT-Zuni populations. The genome-wide data suggests a low level of introgression of *S. aberti kaibabensis* alleles into the ancestors of these populations, which appears to have been accompanied by some introgression of mitochondrial haplotypes, but without complete mitochondrial capture. Under the alternative scenario, introgression of eastern mitochondrial haplotypes into *S. aberti chuscensis* and *S. aberti aberti* MT-Zuni would have needed to occur, which is not supported by genome-wide data. The only piece of evidence seemingly at odds with the direction of introgression we infer is that current *S. aberti kaibabensis* haplotypes might be expected to show characteristics associated with assumed ancestral haplotypes in the haplotype network (e.g. many connections to other nodes; fewer steps from eastern and Mexican haplotypes) since presumably all *S. aberti aberti* and *S. aberti chuscensis* haplotypes would have been derived from ancestral *S. aberti kaibabensis* haplotypes. However, if introgression occurred in the distant past, ancestral haplotypes may have since been lost from the relatively small *S. aberti kaibabensis* population, while being retained in relatively large *S. aberti* populations. Interestingly, the haplotype with the most connections in the western portion of the network is found in both *S. aberti aberti* Coconino-Gila and *S. aberti chuscensis*, which would be consistent with ancient introgression and subsequent retention of *S. aberti kaibabensis* mitochondrial haplotypes into the common ancestor of these populations, a scenario supported by the TREEMIX analysis of the genome-wide data. Altogether, while we cannot completely rule out the alternative scenario, the weight of evidence strongly suggests introgression of *S. aberti kaibabensis* mitochondrial DNA into other western populations rather than the other way around.

Complete mitochondrial capture has been observed in other systems, sometimes even with no evidence for substantial nuclear introgression [13, 30]. Several potential explanations have been proposed to explain this phenomenon, including strong positive selection on favorable mitochondrial haplotypes, sex-related asymmetries in gene flow, and demographic scenarios involving the capture of foreign haplotypes by rapidly expanding populations [3, 11, 13, 14]. Although the explanation in this system is unclear, the fact that complete capture occurred in only one of three populations for which there is evidence of mitochondrial introgression makes selection a less likely explanation. Therefore, we hypothesize that demographic factors or sex-related biases in gene flow most likely explain the patterns we observed, though future work is necessary to provide strong support for this hypothesis. Whatever the explanation for mitochondrial capture, in this case introgression is also evident for nuclear markers, albeit to a lower extent.

In addition to post-divergence gene flow between *S. aberti kaibabensis* and western *S. aberti* populations, we also detected introgression in three additional cases: (1) a population equally related to *S. aberti aberti* Carson-SFW/San Juan and *S. aberti ferreus* Carson E into *S. aberti aberti* Carson-SFW, (2) *S. aberti ferreus* Pike into *S. aberti ferreus* Carson E, (3) *S. aberti barberi* into *S. aberti aberti* Coconino-Gila. While the first case is difficult to evaluate since the source population of introgressed DNA was not sampled, the other cases are generally supported by four population tests and genetic clustering analyses. Introgression involving the two *S. aberti ferreus* populations makes sense geographically, given that these populations are currently connected by corridors of suitable habitat. Introgression of *S. aberti barberi* DNA into *S. aberti aberti* Coconino-Gila highlights the apparent dynamic history of range expansions and contractions of *S. aberti* populations, as these subspecies are currently isolated by substantial areas of unsuitable habitat. Interestingly, although mitochondrial introgression might not be obvious in the case involving *S. aberti ferreus* given the close relationship of the populations, there was no evidence for mitochondrial introgression for the admixture event involving *S. aberti barberi*, which should have been detectable had it occurred. A pattern of nuclear introgression without evidence for mitochondrial introgression is somewhat atypical, as mitochondrial DNA is generally assumed to introgress more easily than nuclear DNA [3, 10, 11]. However, nuclear introgression is more difficult to detect with only a handful of genetic markers [30, 31], so this assumption may be challenged as more genome-wide datasets are generated in the future. Given that nuclear introgression has potentially important consequences for adaptation and speciation [3–6], future work aimed at identifying genomic regions that have introgressed between Abert’s squirrel populations is necessary to elucidate the relative roles of selective and neutral evolutionary forces in driving the patterns we observed.

### Evolutionary relationships and genetic diversity

While we found evidence for gene flow during periods of secondary contact for some groups, overall our data suggest that the evolutionary history of Abert’s squirrels has been largely shaped by divergence during periods of habitat isolation. This is evidenced by the fact that all sampled populations were monophyletic in the phylogenetic analysis, except for the Carson-SFW and San Juan populations, which were intermingled. Based on these data, *S. aberti barberi* from Mexico is the earliest branching lineage in the complex, and the most genetically diverse. This suggests that population sizes have been more stable in the most southern regions occupied by *S. aberti*. A similar split is seen in pines, as *P. arizonica* from the Sierra Madre in Mexico is now considered to be a separate species rather than a southern variety of ponderosa pine [32]. Previous estimates based on mitochondrial substitution rates suggest that the split between *S. aberti barberi* and other populations occurred approximately 1–1.5 million years ago [21, 22]. Based on similar relative levels of divergence, *S. aberti kaibabensis* appears to have separated from other northern populations around the same time. It is not clear, however, when *S. aberti kaibabensis* reached the north rim of the Grand Canyon. Based on the paleoecological record, ponderosa pine was previously assumed to be absent from areas north of the 36th parallel (central Arizona and New Mexico) during the last glacial maximum [33–35]. If true, this would imply that *S. aberti* arrived on the north rim relatively recently. Given the amount of genetic divergence that has accumulated since *S. aberti kaibabensis* split from other *S. aberti*, this would suggest that substantial divergence would have had to occur prior to reaching the north rim. However, recent genetic analyses of ponderosa pine indicate that glacial refugia were likely to have been present in more northern regions as well [36, 37]. Moreover, climate niche modeling indicates a high probability of occurrence for the western variety of ponderosa pine (*P. ponderosa* var. *ponderosa*) during the last glacial maximum (~ 22,000 yr. BP) in the area currently occupied by *S. aberti kaibabensis* [38]. Although *S. aberti* is not currently associated with this variety of ponderosa pine, this does not rule out the possibility that such associations existed in the past. If so, *S. aberti kaibabensis* may have persisted in areas north of the Grand Canyon for longer than previously assumed. Altogether, our data support earlier interpretations of *S. aberti kaibabensis* as a highly distinct lineage endemic to a remarkably small area of habitat (~ 89,000 ha) on the north rim of the Grand Canyon [39]. Low levels of genetic variation relative to all other populations, and the diminutive range of *S. aberti kaibabensis* implicate genetic drift in playing a large role in facilitating divergence between this subspecies and other *S. aberti* populations.

For the remaining *S. aberti* populations, although some caution is warranted in interpreting the relationship between *S. aberti aberti* Coconino-Gila and other populations (see results), the bulk of the evidence suggests a subsequent split between western and eastern groups followed by further splits within each clade. The overall significant level of differentiation among all *S. aberti* populations of Colorado, New Mexico, and Arizona stands in contrast to relatively lower levels of differentiation among ponderosa pine populations from these same areas, at least as revealed by studies using a handful of nuclear and mitochondrial genetic markers [56, 57]. Although these studies did provide some evidence for differentiation between northern and southern ponderosa pine populations (roughly corresponding to areas occupied by *S. aberti ferreus* and *S. aberti aberti*), the magnitude of this differentiation was relatively low compare to that observed in *S. aberti*. Areas of unsuitable habitat separating *S. aberti* populations thus appear to be more formidable barriers to current gene flow among squirrel populations, as divergence even among neighboring populations is relatively high. This is somewhat surprising as previous studies have documented the rapid spread of introduced Abert’s squirrel populations into new areas even when separated by patches of unsuitable habitat [40]. In Colorado, populations from eastern and western portions of the state are genetically isolated even though areas of suitable habitat connect them (Fig. 1). Although ponderosa pine is sparse in these connecting corridors, Abert’s squirrels are known to be present. However, previous authors have noted that these areas appear to have been colonized during a recent range expansion, which could explain the lack of a significant genetic signature of admixture between eastern and western lineages outside of these connecting corridors [41]. Future sampling in these corridors may thus reveal evidence for admixture, similar to what is observed in the *S. aberti ferreus* Carson E population.

Like previous genetic studies, our results provide support for some, but not all, currently *S. aberti* subspecies designations. Although there is no formal genetic metric for distinguishing subspecies, our data confirm that *S. aberti barberi* and *S. aberti kaibabensis* are the earliest diverging lineages in the group and are highly distinct from other *S. aberti*. Nevertheless, evidence for gene flow between these and other *S. aberti* populations since divergence suggests that reproductive isolation between these and other populations might be absent or incomplete despite considerable genetic divergence. While challenging, future studies assessing the potential for reproductive isolation between the subspecies (*S. aberti barberi* and *S. aberti kaibabensis*) and the rest of the group, would be necessary to determine whether these populations are isolated enough to be considered different species, as has been argued in the past (e.g. earlier schemes designated *S. kaibabensis* as a separate species). Moreover, future studies genetic studies aimed at determining when gene flow from *S. aberti kaibabensis* and *S. aberti barberi* into other populations occurred would be informative. Our data conflicts to some degree with current subspecies designations for the rest of the group, with the most obvious discrepancy involving *S. aberti aberti*. According to the last formal taxonomic revision of *S. aberti* [20], this subspecies includes populations from Arizona south of the Grand Canyon, western New Mexico (e.g. the Mount Taylor and Zuni Mountains), southwestern Colorado (San Juan), and northern New Mexico (Carson-SFW). However, our genome-wide data confirm previous conclusions from mitochondrial studies suggesting that Carson-SFW and San Juan populations are most closely related to *S. aberti ferreus* (Pike and Carson E in this study) [21, 22]. Furthermore, our data indicate that *S. aberti aberti* MT-Zuni is more closely related to *S. aberti chuscensis* than *S. aberti aberti* from Arizona. It is thus clear that *S. aberti aberti*, as currently described, does not accurately reflect the evolutionary relationships among the included populations. Our data also provide strong support for an eastern clade that includes populations from Pike, Carson E, San Juan, and Carson-SFW, which is consistent with previous suggestions that the range of *S. a ferreus* should be expanded to include populations in western Colorado and northern New Mexico [21, 22]. Within this clade, there is relatively strong differentiation between Pike and San Juan/Carson-SFW, with Carson E being highly admixed between these two lineages. Future sampling in corridors connecting populations in eastern and western Colorado will provide insight into whether further admixture occurs in this region. Unfortunately, although mitochondrial data suggested that *S. aberti barberi* and *S. aberti durangi* might not be genetically distinct [21, 22], we were not able to obtain enough samples of *S. aberti durangi* to evaluate the relationship between these subspecies.

### Genetic diversity and future response to climate change

Consistent with inferences from mitochondrial data [21, 22], with the exception of *S. aberti kaibabensis*, current range sizes for *S. aberti* subspecies do not correlate strongly with observed patterns of genetic diversity. For example, despite the small range size of *S. aberti chuscensis* this population harbors relatively high levels of genetic variation. Moreover, despite large areas of contiguous ponderosa pine habitat in Colorado, levels of genetic diversity in these populations were relatively low. In general, genetic diversity in *S. aberti* declines moving from the south to the north and east. This is consistent with initial expansion from southern glacial refugia, as has been proposed by previous authors [40], followed by subsequent colonization of areas further to the north and east. However, we also note new evidence indicating the likely presence of more northern/eastern refugia for ponderosa pine [36–38]. If these refugia were comparatively smaller and therefore harbored less genetic diversity, then a scenario involving divergence through vicariance rather than colonization might also explain observed patterns of genetic diversity.

It is unclear to what degree more recent human-based activities such as timber harvesting, fire suppression, grazing, and hunting have influenced observed levels of genetic diversity. The impact of such practices on squirrel populations is potentially substantial, as previous studies have demonstrated that forest structure has important effects on the quality and suitability of Abert’s squirrel habitat [42–45]. Variability in the intensity of these factors across the range of Abert’s squirrels may thus also contribute to observed differences in relative levels of genetic diversity among populations.

As a highly specialized herbivore dependent almost entirely on ponderosa pine, *S. aberti* is likely to be greatly impacted by future climate change. Predictive models suggest that a large portion of the area currently occupied by Abert’s squirrels will be unsuitable habitat for ponderosa pine in the coming decades [37]. Given that Abert’s squirrels currently occupy only a small portion of the total range of ponderosa pine, future studies that combine niche modeling specifically for Abert’s squirrels under different scenarios of climate change are necessary in order to fully understand the likely impact of habitat loss on Abert’s squirrel populations. Risks associated with future habitat loss are further underscored by the fact that much of the current genetic diversity in the Abert’s squirrel is distributed among relatively small stands of ponderosa pine that are highly isolated. For example, our study reveals that *S. aberti kaibabensis* is a highly distinct genetic lineage currently occupying an area encompassing only about 89,000 ha [39]. Abert’s squirrel have a complex relationship with ponderosa pines involving both negative impacts on individual plant fitness through selective herbivory [46], and positive impacts through dispersal of spores from mycorrhizal fungi on which trees depend [47–49]. Moreover, digging activity of Abert’s squirrels has numerous positive effects on the forest ecosystem by creating microhabitats for other organisms, and redistributing nutrients and water into tree root zones [50]. Impacts of future climate change on squirrels and/or ponderosa pine may therefore have far-reaching consequences for forest communities.

## Conclusions

The results or our study suggest that Abert’s squirrels have a complex evolutionary history involving divergence in isolation with subsequent gene flow occurring between some populations during periods of secondary contact. This interbreeding resulted in discordant patterns of introgression across mitochondrial and nuclear genomes, with mitochondrial introgression being higher than nuclear introgression in some cases and lower in another. These variable outcomes suggest that large genome-wide datasets may be necessary to accurately assess the extent of mito-nuclear discordance. Overall, our results highlight the utility of large genome-wide datasets for inferring the evolutionary history of species that have diversified under complex scenarios involving divergence with gene flow.

## Methods

### Sample collection and preparation

We used DNA samples originally collected as part of previous genetic studies of the Abert’s squirrel species complex [21, 22, 51–53], and also obtained 14 additional tissue samples from the Museum of Southwestern Biology, and one *S. griseus* tissue sample from The Museum of Vertebrate Zoology at Berkeley for use as an outgroup. We follow subspecies designations as proposed by Hoffmeister & Diersing [20] to describe sample populations. Samples included in this study represent five of the six currently recognized subspecies [20]. We did not include *S. aberti durangi* from Mexico, as only one sample was available for this subspecies, which would preclude population genetic inferences. Approximate collecting locations and sample sizes are shown in Fig. 1, and detailed location information is given in Additional file 2. Genomic DNA from samples used in earlier studies were prepared as previously described [21, 22, 51–53]. Museum samples (spleen or liver) were preserved in ethanol and DNA was extracted using the Qiagen DNeasy Blood and Tissue kit following manufacturer’s protocols. Given previous results from phylogeographic studies based on mitochondrial DNA samples that indicated mixing of divergent eastern and western haplotypes in the range of *S. aberti chuscensis* [21, 22], we genotyped individuals of this subspecies prior to analysis to ensure we had sampled 10 individuals carrying each of the mitochondrial haplotypes. Specifically, we designed a restriction fragment length polymorphism assay based on published *cytb* sequences [21]. We used the restriction enzyme *HphI*, which detects different polymorphisms in *cytb* haplotypes. Prior to library preparation, all genomic DNA samples were run on agarose gels to verify the presence of abundant high molecular weight DNA with little degradation. A total of 95 genomic DNA samples were sent to Floragenex, where Restriction Site Associated DNA (RAD) Illumina libraries were prepared using *SbfI* as the restriction enzyme and sequenced on an Illumina Hiseq2000 with a 100 bp single end protocol.

### RAD-seq data processing

Sequencing yielded a total of 144,307,453 reads across the 95 samples. Data were processed using STACKS version 1.37 [54, 55] . Sequences were first demultiplexed and cleaned using the *process_radtags* script with default settings, which resulted in a total of 137,353,563 clean reads across the 95 libraries (mean = 1,445,827; range: 29,605 – 3,095,410). The sample with the lowest number of reads was removed from subsequent analyses due to large amounts of missing data, and we also removed two additional samples that had been mislabeled (these samples are not included in sample sizes given in Fig. 1). We ran the STACKS pipeline using the *denovo_map.pl* wrapper on two separate datasets: (1) all *S. aberti* samples and the *S. griseus* outgroup sample, and (2) all *S. aberti* samples. We used the default minimum stack depth (−m) of three, which is the number of identical reads required to initially form a stack (roughly corresponding to an allele). We ran a range of different parameter settings for distance allowed between stacks (−M 2–3) and distance between catalog loci (−n 3, 5, 7, 9 for the dataset with the outgroup and –n 2, 3, 5 for the dataset with no outgroup). The distance allowed between stacks parameter (−M) corresponds to the maximum number of nucleotide differences between stacks allowed for them to be merged into a single locus (e.g. -M 2 would mean alleles at a single locus in an individual could not differ by more than two nucleotides). Once loci are built in all individuals, the data from each individual is used to generate a catalog representing all the alleles and loci in the dataset (in this case across all sampled populations). The distance between catalog loci parameter (−n) sets the maximum number of mismatches between catalog loci for them to be merged into a single locus. For example, if -n is set to two then loci from different individuals differing by two or fewer nucleotides would be merged into a single locus. We ran all resulting datasets through the stacks corrections module (*rxstacks*) using the following parameter settings: --lnl_limit − 10, −-prune_haplo, −-model_type bounded, −-bound_high 0.05, −-conf_lim 0.2. We compared *F*-statistics computed by the STACKS *populations* program for each of the parameter set combinations to assess the impact of different parameter settings on the resulting datasets. In general, we did not find major differences across the range of parameter settings although the total number of identified loci varied. Considering this, we chose to use -M 3, −n 5 for the dataset with the outgroup, and –M 2, −n 3 for the dataset with no outgroup.

### Genetic diversity and population structure

To extract SNPs for population genetic analyses we used the *populations* program in STACKS. We used the *write_single_snp* option to include only one SNP per RAD locus since most downstream analyses assumed loci are unlinked. We included loci that were present in at least 50% of individuals in 7/10 groups (nine localities with two separate groups representing the divergent mitochondrial lineages in *chuscensis*), which yielded a total of 67,430 loci. Population structure was analyzed in several ways. Pairwise *F*_*ST*_ values were estimated between all groups using the R package ASSIGNER [56], which calculates Weir and Cockerham’s pairwise *F*_*ST*_ values [57] and their confidence intervals. The haplotype file generated by the *populations* program in STACKS was used as input for this analysis. We also performed Principal Component Analysis (PCA) using the R package SNPRelate [58]. We used the software programs ADMIXTURE version 1.3.0 [59] and STRUCTURE version 2.3.4 [60] to infer the optimal number of genetic clusters (*K*) present in the data and produce estimates of individual ancestry from each cluster. These programs differ in that ADMIXTURE uses a maximum likelihood approach while STRUCTURE relies on Bayesian methods. We used the full dataset for ADMIXTURE, but because STRUCTURE is less efficient with large datasets, we randomly selected 6000 SNPs for this analysis. For both programs, we ran 20 replicates for *K* values ranging from 1 to 10. For the STRUCTURE analysis, we used the admixture model with correlated allele frequencies, and ran the MCMC simulations for 50,000 generations after a burnin of 10,000 generations. Convergence was assessed by analyzing the values of summary statistics to ensure estimates had stabilized. To choose the optimal *K* value we used the cross-validation (CV) procedure available in ADMIXTURE [59] and Evanno’s Δ*K* method [61] for the STRUCTURE analysis. These analyses were both complicated by the presence of multimodality for several values of *K*, which resulted in highly variable cross-validation (CV) errors or data likelihoods for different modes of a given value of *K*. This made the usual practice of averaging over replicates of *K* problematic. To deal with this issue, we used the software PONG [62] to formally identify different modes for each value of *K* using a similarity threshold of 0.97 to group replicates into the same mode. Once different modes were identified, we retained only the best mode for each value of *K* for further analysis (i.e. lowest CV error for ADMIXTURE or highest likelihood for STRUCTURE). In ADMIXTURE, we then chose the optimal *K* based on the lowest average CV error across replicates. For structure, we used Evanno’s Δ*K* method [61] as implemented in STRUCTURE HARVESTER version 0.6.94 [63].

Genetic diversity estimates (observed heterozygosity, *π*, and number of polymorphic loci) were obtained using the –*fstats* option in the *populations* program in STACKS. Because analyses of population structure failed to detect any structure between samples carrying the divergent mitochondrial lineages present in the *S. abert chuscensis* subspecies (see results), we combined these samples into one group for genetic diversity estimates.

### Evolutionary relationships and introgression among populations

The dataset with the outgroup (*S. griseus*) was used for phylogenetic analysis. Given computational demands and to reduce the amount of missing data, we included 3–4 individuals from each of the sampled localities (including four samples from each mitochondrial lineage in *S. a. chuscencis*) and the one outgroup sample (40 individuals total) in the analysis. The individuals from each population were chosen because of their relatively high sequencing coverage. We used the *populations* program in STACKS to generate an alignment file including concatenated loci present in at least 30/40 individuals (20,792 loci total). We used JMODELTEST2 [64] with Akaike Information Criterion (AIC) and Bayesian Information Criterion (BIC) model selection procedures to choose an appropriate nucleotide substitution model. These model selection procedures provided the strongest support for two nucleotide substation models: (1) General Time Reversible (GTR) model (second highest model using both BIC and AIC selection procedures, and (2) Transversion model (TVM) (ranked first with BIC and third with AIC). We built maximum-likelihood phylogenetic trees using each nucleotide substitution model with RAXML-NG [65, 66]. We applied Lewis’s ascertainment bias correction [67] as recommended for concatenated SNP datasets [28], with 200 bootstrapping replicates to evaluate branch support.

Given that genetic clustering analyses suggested a history of admixture between some populations, a bifurcating phylogenetic tree may not fully represent the evolutionary history and/or relationships of the sampled populations. We therefore used the program TREEMIX ver. 1.12 [68] to investigate the history of population divergence and gene flow between populations. TREEMIX uses allele frequencies and a Gaussian approximation of genetic drift among populations to first estimate a maximum-likelihood bifurcating tree. Migration edges are then inferred in a step-wise manner based on residuals from the covariance matrix indicating a poor fit to the tree model for pairs of populations. We used the population genetic dataset that combined all the *S. aberti chuscensis* samples (see above) for this analysis. We designated *S. aberti barberi* as the outgroup given the results of phylogenetic analysis by RAXML-NG, which confidently assigned *S. aberti barberi* as the earliest branching lineage in the *S. aberti* complex. We ran TREEMIX with 0–6 migration edges using the –global option to perform a global rearrangement. We performed five runs for each migration model using a different random seed to assess the consistency of the results. We used a function available in the RADpipe package [69] for R to calculate the percentage of variance explained by models incorporating different numbers of migration edges. Although TREEMIX produces *p*-values for migration edges, the authors caution that these values may be unreliable estimates of significance since they are generated under a heavily parameterized model [68]. As suggested by the authors, we therefore used less parameterized four-population tests to evaluate the significance of individual migration events indicated by TREEMIX. The four-population test can be used to distinguish incomplete lineage sorting and introgression using allele frequency data from four populations [70]. Assuming an unrooted tree ((A,B),(C,D)), under incomplete lineage sorting alone allele frequency differences between A and B should be independent of differences between C and D, and the *f*_*4*_ statistic should be zero. However, a positive *f*_*4*_ value would indicate introgression between populations A and C or B and D, while a negative *f*_*4*_ value would indicate introgression between A and D or B and C. This test is highly robust to different demographic scenarios and is therefore a powerful test of introgression. We conducted four-population tests using the program *F4* [71], which tests the significance of *f*_*4*_ values using coalescent simulations to assess the probability that the observed results could be produced by incomplete lineage-sorting alone. Since positive and negative *f*_*4*_ values do not necessarily discriminate between two possible scenarios of introgression (e.g. a positive value could result from introgression from A to C or B to D), we set-up our tests with trees that made one of the two scenarios unlikely and, therefore, provide a more direct test for gene flow between a focal population pair.

### Mitochondrial introgression: Construction of *cytb* haplotype network and haplotyping

To visualize relationships among *cytb* haplotypes generated by a previous study [21], we downloaded haplotype sequences from GenBank (AAB00562 – AAB00587) and built a TCS haplotype network [72] using POPART software [73]. We note that a few of the sequences from GenBank (AABB05569- AAB05573) included three additional nucleotides toward the 5′ end of the gene that were not represented in the alignment presented in Wettstein et al. [21] but are also present in sequences from other *Sciurus*. Given this discrepancy, we excluded these nucleotides from all sequences in our analysis. In previous studies, all samples from Coconino-Gila and *S. aberti kaibabensis* had haplotypes from the western lineage, all samples from MT-Zuni had eastern haplotypes, while *S. chuscensis* had representatives from both lineages [21, 22]. To further examine the distribution of haplotypes in these areas we genotyped additional samples from each location and from Cibola National Forest (just east of Gila National Forest) using the restriction profiling strategy described above (*S. aberti* Coconino-Gila, *N* = 44; *S. aberti* Cibola, *N* = 11; *S. aberti kaibabensis*, *N* = 47; MT-Zuni, *N* = 7; *S. aberti chuscensis*, *N* = 51). These DNA samples were from previous collections or were taken from tissue samples supplied by the Museum of Southwestern Biology (Additional file 2).

## Additional files

Additional file 1: Pairwise F_*ST*_ and confidence intervals. (XLSX 50 kb)

Additional file 2: Collecting information and *cytb haplotyping* results. Tab one includes detailed information on the collecting locations of each sample and museum identifiers when applicable. Tab two includes the results of *cytb* haplotyping. (XLSX 20 kb)
